# Synergistic Effects of Various Ceramic Fillers on Thermally Conductive Polyimide Composite Films and Their Model Predictions

**DOI:** 10.3390/polym11030484

**Published:** 2019-03-13

**Authors:** Heeseok Song, Byoung Gak Kim, Yong Seok Kim, Youn-Sang Bae, Jooheon Kim, Youngjae Yoo

**Affiliations:** 1 Division of Advanced Materials, Korea Research Institute of Chemical Technology, Daejeon 34114, Korea; song1918@krict.re.kr (H.S.); bgkim@krict.re.kr (B.G.K.); yongskim@krict.re.kr (Y.S.K.); 2 Department of Chemical and Biomolecular Engineering, Yonsei University, Seoul 03722, Korea; mowbae@yonsei.ac.kr; 3 Department of Chemical Convergence Materials, University of Science and Technology, Daejeon 34113, Korea; 4 School of Chemical Engineering & Materials Science, Chung-Ang University, Seoul 156-756, Korea

**Keywords:** thermal conductivity, binary filler, modeling, polymer composite

## Abstract

In this study, thermally conductive composite films were fabricated using an anisotropic boron nitride (BN) and hybrid filler system mixed with spherical aluminum nitride (AlN) or aluminum oxide (Al_2_O_3_) particles in a polyimide matrix. The hybrid system yielded a decrease in the through-plane thermal conductivity, however an increase in the in-plane thermal conductivity of the BN composite, resulting from the horizontal alignment and anisotropy of BN. The behavior of the in-plane thermal conductivity was theoretically treated using the Lewis–Nielsen and modified Lewis–Nielsen theoretical prediction models. A single-filler system using BN exhibited a relatively good fit with the theoretical model. Moreover, a hybrid system was developed based on two-population approaches, the additive and multiplicative. This development represented the first ever implementation of two different ceramic conducting fillers. The multiplicative-approach model yielded overestimated thermal conductivity values, whereas the additive approach exhibited better agreement for the prediction of the thermal conductivity of a binary-filler system.

## 1. Introduction

Heat generation in electronic devices has a significant effect on the performance of these devices; thus, various methods of thermal control have recently attracted considerable attention. To solve the heat dissipation problem in electronic devices, polymeric hybrid materials, such as alumina (Al_2_O_3_) [1,2,3] silicon carbide (SiC) [4], aluminum nitride (AlN) [5], and hexagonal boron nitride (BN) [6,7], have been used as thermally conductive ceramic fillers in polymer matrices. Also, graphene is used to improve the thermal conductivity of composites. Zhaid et al. reported the graphene supported thermal interface material (TIM), which has outstanding thermal and electrical conductivity by a simple fabrication process [8].

Among these materials, BN seems the most promising, owing to its high thermal conductivity (up to 400 W/m∙K) and relatively low dielectric constant (approximately four), compared with those of other ceramic fillers. Moreover, the thermal conductivity of BN depends on the orientation direction (i.e., is anisotropic), thereby leading to a high versatility of BN in product design [9,10]. Thermally conductive composites containing ceramic fillers in epoxy [2], high-density polyethylene (HDPE) [11], linear low-density polyethylene (LLDPE) [12], polystyrene (PS) [13], and polyamide (PA) [6] have been previously reported. Recently, polyimide (PI)-based composite materials with outstanding properties (high chemical resistance, high mechanical strength, high thermal stability, and low dielectric constants) have been widely applied to packaging and insulating materials in the microelectronics and aerospace industries. Unfortunately, high concentrations of conducting filler in these materials hinder the use of the composites in various applications, owing to the loss of polymeric material [6,7]. Several studies have considered binary filler systems for polymer composites. Che et al. fabricated high-density polyethylene/boron nitride/carbon nanotubes (CNTs) via melt-mixing and subsequent hot rolling and reported that as the content of BN increases, BN forms an effective network with CNTs in the matrix [14]. Bian et al. prepared dopamine modified binary filler, micro-sized BN, and nano-sized Al_2_O_3_ for epoxy composites. They confirmed that BN mainly built the heat conduction network and Al_2_O_3_ formed a bridge between the BN particles [14]. Chen et al. [15] fabricated epoxy composites with two types of spherical Al2O3 to control the viscosity and thereby improve the processability; these composites exhibited high thermal conductivity. Furthermore, Choi et al. [16] prepared Al_2_O_3_ and AlN of different sizes with the aim of improving the packaging loading of fillers, and the resulting composites yielded high thermal conductivity.

As an extension of a previous study based on carbon fillers [17], the present work represents a systematic investigation of the morphology and properties characterizing composite films based on ceramic fillers. The effects of individual and multiple fillers on the morphology and thermal conductivity are discussed. Furthermore, a detailed model prediction for the composites is presented and used for the incorporation of two fillers.

## 2. Experimental

### 2.1. Materials

Analytical grade pyromellitic dianhydride (PMDA) and 4,4′-oxydianiline (ODA) were obtained from Daicel Chemical (Osaka, Japan) and Wakayama Seika Kogyo Co. Ltd. (Wakayama, Japan). *N*,*N*-dimethylacetamide (DMAc) solvent was purchased from J.T. Baker (Phillipsburg, NJ, USA). Preferentially, PMDA was purified by vacuum sublimation. The BN powder (with an average particle size of approximately 5 µm) was purchased from ChangSung (Seoul, Korea), while the Al_2_O_3_ (average particle size > 10 µm) and AlN (average particle size of approximately 10 µm) powders were acquired from Sigma Aldrich (St. Louis, MO, USA).

### 2.2. Preparation of Polyimide-Filler Composite Films

**Scheme 1:**
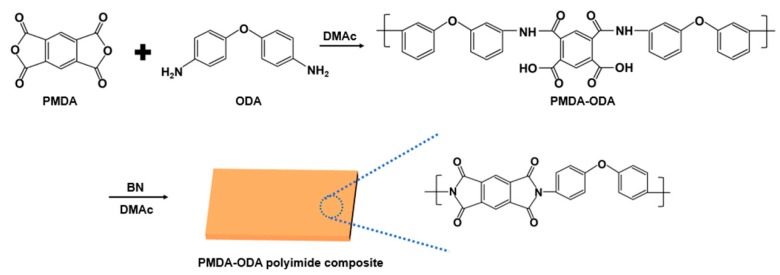
Illustration of the PI (polyimide) composite fabrication procedure.

The PMDA powder was first purified by vacuum sublimation. First, a specified amount of 4,4′-oxydianiline (ODA) was stirred with *N*,*N*-dimethylacetamide (DMAc) solvent. Then, a pyromellitic dianhydride (PMDA) of the same molar ratio as 4,4′-oxydianiline (ODA) was added. Therefore, a PMDA-ODA (polyamic acide, PAA) precursor of 20 wt % was prepared. A specified amount of the BN filler mixture with 3.3 mL of DMAc was added with 10 g of prepared PAA under N_2_ atmosphere and was agitated overnight using magnetic stirring. The weight ratios of the hybrid BN+AlN and BN + Al_2_O_3_ fillers that were utilized in this study were equal to 1:1. The filler contents were manufactured with 10, 20, and 30 wt %. The prepared samples were first cast on a 230 × 85 × 3 mm glass plate using a doctor blade and were then dried at 40 °C in a vacuum oven for 4 h to remove solvent traces as well as any micro- or nano-sized bubbles. After that, the samples were polyamic acid, PAA subjected to imidization in a convection oven at 120, 180, and 250 °C for 30 min and then at 350 °C for 1 h. The fabrication procedure of PI composites is illustrated in Scheme 1.

### 2.3. Characterization

The cross-sectional fracture surfaces of the film were polished using an ion polishing system (Ilion II model 697, Thomson Scientific Instruments, Box hill, Victoria, Australia). To investigate the morphology of the fillers and PI matrices, the cross-sectional fracture surfaces of the films were observed by using field emission scanning electron microscopy (FE-SEM, Merlin, Carl Zeiss, Oberkochen, Germany) at the Korea Basic Science Institute, Daejeon center (KBSI). Also, thermal diffusivity was measured at room temperature using a laser flash analyzer (LFA 447, Netzsch, Waldkraiburg, Germany). Then, the densities and specific heats of the film were measured by a gas pycnometer (AccuPyc Ⅱ 1340, Micromeritics, Norcross, GA, USA) and differential scanning calorimeter (DSC Q200, TA instruments), respectively. The thermal conductivity (*k*) values were calculated using a heat conduction equation that describes the relationship between thermal diffusivity (*α*), density (*ρ*), and specific heat (*C_p_*) of the polymer (*k = α∙ρ∙C_p_*).

## 3. Results and Discussion

### 3.1. Morphology

**Figure 1:**
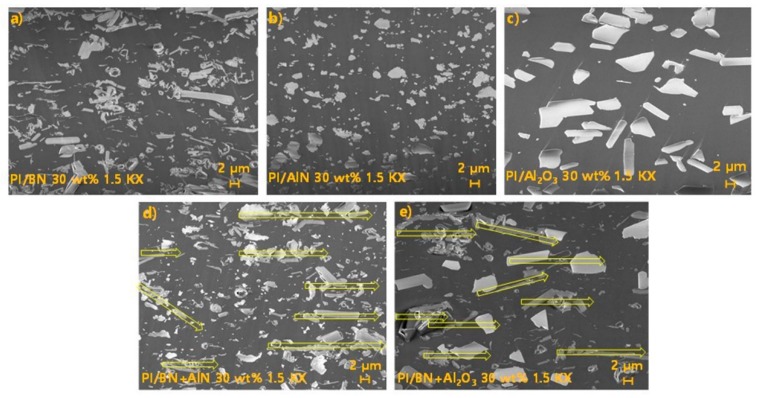
FE-SEM (field emission scanning electron microscopy) images of the cross-sectional fracture surfaces of the composite films containing (**a**) 30 wt % of PI/BN (boron nitride); (**b**) 30 wt % of PI/AlN (aluminum nitride); (**c**) 30 wt % of PI/Al_2_O_3_; (**d**) 30 wt % of PI/BN+AlN (1:1); and (**e**) 30 wt % of PI/BN+Al_2_O_3_ (1:1) (the arrow mark meaning the effective thermal pathway originated from the connection of thermal conductive fillers).

All samples that were used in this study exhibited a high degree of flexibility, up to 30 wt % concentration of the filler, regardless of the filler type. The morphology of the cross-sectional fracture surfaces was investigated via FE-SEM. Figure 1a shows a FE-SEM image of the PI composite film containing 30 wt % BN. The BN filler consists of plate-shaped particles which are aligned primarily along the horizontal direction of the film. Images of the films containing 30 wt % AlN, 30 wt % Al2O3, 30 wt % BN+AlN (1:1), and 30 wt % BN+Al2O3 (1:1) fillers (Figure 1b–e, respectively) indicate a significant degree of interaction between different filler particles. At small filler amounts, alignment of the plate-shaped BN particles primarily along the horizontal direction of the composite films prepared via casting is difficult, owing to the relatively low shear force applied to the films. However, the morphology shown in Figure 1d,e reveals that the filler particles will probably interact in the horizontal direction. Therefore, the in-plane thermal conductivity increased, owing to the well-connected horizontal heat flow path generated by the binary filler system.

### 3.2. Thermal Conductivity

**Figure 2:**
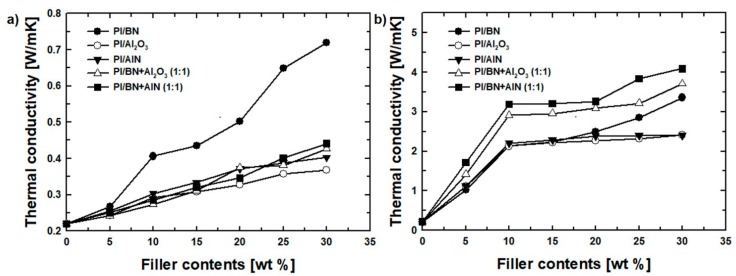
Thermal conductivity along the (**a**) through-plane and (**b**) in-plane directions of the composite films plotted as functions of the filler content.

**Figure 3:**
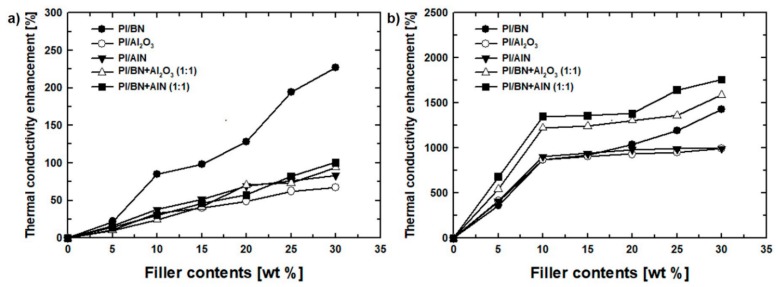
Thermal conductivity enhancements along the (**a**) through-plane and (**b**) in-plane directions of the composite films plotted as functions of the filler content.

Figure 2a,b show the values of thermal conductivity calculated along the through-plane and in-plane directions, respectively, of the obtained films. Figure 3a,b show the corresponding enhancements of the conductivity along these orientations, with the highest values occurring along the in-plane direction of the films containing hybrid fillers. The maximum (4.091 W/m∙K) and minimum (2.404 W/m∙K) values of the thermal conductivity along the in-plane direction were observed for the samples with 30 wt % PI/BN+AlN and the sample with 30 wt % AlN, respectively. However, the conductivities along the through-plane direction were larger for the films containing only the BN filler than for the composites with hybrid fillers. A maximum value of 0.719 W/m∙K was observed for the sample with 30 wt % of BN and a minimum value of 0.368 W/m∙K occurred for the film containing a mixture of BN and Al_2_O_3_. The through-plane thermal conductivity results show that BN is a better thermally conductive filler than Al_2_O_3_ and AlN for the PI-based composite film. Therefore, the reduction in the BN content and addition of other filler leads to thermal conductivity degradation. However, compared with the Al_2_O_3_ and AlN composites, the hybrid filler system exhibited lower through-plane thermal conductivity, however higher in-plane thermal conductivity. These behaviors can be attributed to the interaction between the filler particles described in the previous section. The hybrid fillers lead to increased filler alignment along the in-plane direction, thereby obstructing the through-plane thermal pathways. As a result, added Al_2_O_3_ or AlN leads to increased interaction between the filler and an extra thermal pathway along the in-plane direction.

### 3.3. Modeling

Various model equations describing the relations among the aspect ratio, density, filler shape and type, modulus, orientation, thermal conductivity, and weight fraction have been proposed for predicting the effective thermal conductivity of polymer composites [18,19,20,21,22,23]. The regular and modified Lewis–Nielsen models are generally used for this prediction. In this work, the results calculated by using these models were compared with the obtained experimental data. The main equation governing these models may be expressed as follows [24,25,26,27]:

(1) $$K=k_{m}\cdot \left[\frac{1+A\cdot B\cdot \phi_{f}}{1-B\cdot \phi_{f}\cdot \Psi }\right]$$

where $A=k_{E}-1$ and $B=\frac{k_{f}/k_{m}-1}{k_{f}/k_{m}+A}$; $k_{E}$, $k_{m}$, $k_{f}$, and $\phi_{f}$ are the Einstein coefficient, thermal conductivity of a polymer matrix, thermal conductivity of a composite, and packing fraction of a filler, respectively. $\Psi =1+\frac{1-\phi_{max}}{\phi_{max}{}^{2}}\cdot \phi_{f}$ for the Lewis–Nielsen model and $\Psi =1+\frac{\phi_{m}}{\phi_{max}}\left[\phi_{max}\cdot \phi_{f}+\left(1-\phi_{max}\right)\cdot \phi_{m}\right]$ (here, $\phi_{m}$: packing fraction of the matrix and $\phi_{max}$: maximum packing fraction of the filler) for the modified Lewis–Nielsen model.

**Figure 4:**
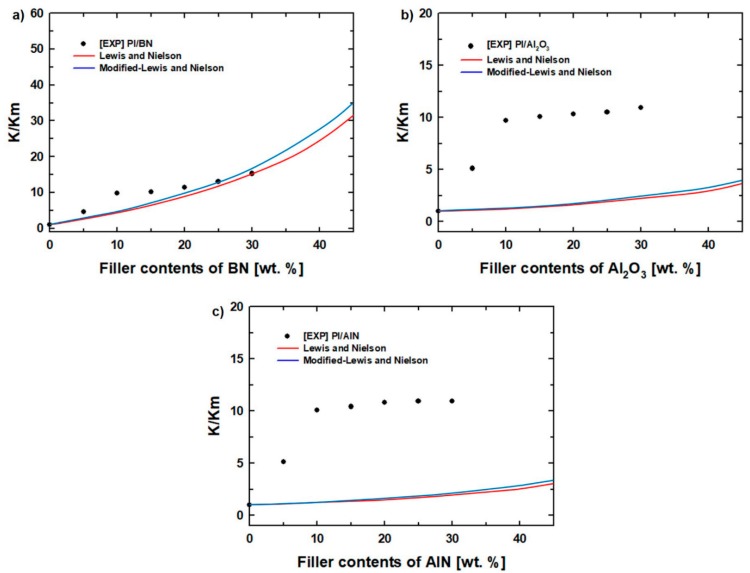
Experimental and theoretical thermal conductivity obtained using the regular and modified Lewis–Nielsen models for the (**a**) PI/BN; (**b**) PI/Al_2_O_3_; and (**c**) PI/AlN composite films.

Figure 4 shows the theoretical prediction and experimental thermal conductivity data for the PI/BN, PI/Al_2_O_3_, and PI/AlN composites. Good fitting between the experimental and theoretical data in both models was realized only for the PI/BN composite. The other composites were considerably underestimated. The theoretical models described above are valid for systems consisting of single fillers inside a polymer matrix. However, the hybrid filler systems utilized in this study were composed of mixtures containing two different fillers, namely BN and Al_2_O_3_ or AlN particles. Therefore, a model that considers two filler types is required for accurate prediction of the experimental results [18,28,29,30]. Two population models involving additive and multiplicative approaches are often utilized for this purpose. The additive approach method can be described by the following equation:

(2) $$\frac{K_{add}}{k_{m}}=\frac{k_{f1}}{k_{m}}+\frac{k_{f2}}{k_{m}}-1$$

**Figure 5:**
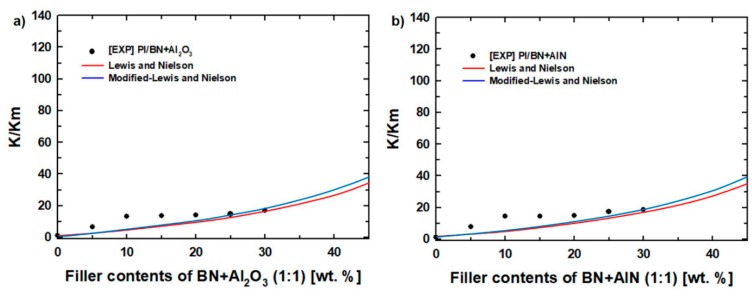
Experimental and theoretical thermal conductivity obtained for the (**a**) PI/BN+Al_2_O_3_ and (**b**) PI/BN+AlN composites by using the additive approach model.

**Figure 6:**
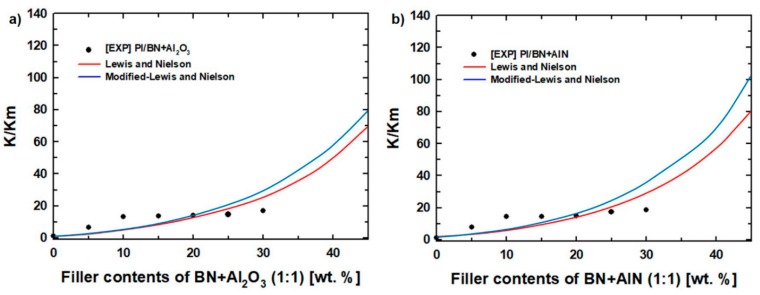
Experimental and theoretical thermal conductivity obtained for the (**a**) PI/BN+Al_2_O_3_ and (**b**) PI/BN+AlN composites by using the multiplicative approach model.

Here, $K_{add}$ is the predicted thermal conductivity of a hybrid filler composite, whereas $k_{f1}$ and $k_{f2}$ are the thermal conductivities of the composites with only the BN- and Al-containing fillers, respectively. The experimental results correspond closely to the theoretical predictions obtained via the additive approach method applied to the regular and modified Lewis–Nielsen models (see Figure 5). In the multiplicative approach method, the contribution of the BN filler to the thermal conductivity of a composite is calculated first and the obtained BN-containing composite is considered the matrix for the second Al_2_O_3_ or AlN filler. In other words, the contribution of the second filler to the thermal conductivity is calculated by using the PI/BN composite matrix ($k_{C}$) rather than the neat PI matrix ($k_{m}$). The obtained contributions of both fillers are multiplied as follows [18,28],

(3) $$\frac{K_{mult}}{k_{m}}=\left(\frac{k_{f2}}{k_{C}}\right)\cdot \left(\frac{k_{f1}}{k_{m}}\right)$$

where ***K_mult_*** is the predicted thermal conductivity of the composite. The additive and multiplicative models are commonly used to predict synergetic filler effects on thermal conductivity. However, when the filler content is relatively high, a greater improvement in the conductivity is often better predicted by using the multiplicative approach method rather than the additive approach. Figure 6 shows the thermal conductivity data predicted by the multiplicative approach method for the composite films and the corresponding experimental values. As Figure 5 indicates, the hybrid-filler thermal conductivity data measured in this study concur more closely with the additive approach applied to the regular and modified Lewis–Nielsen models than with the multiplicative model presented in Figure 6. The measured conductivity and the conductivity predicted from the additional approach increase in a similar manner, whereas, in the multiplicative approach model, the predicted values are overestimated compared with the experimental results. Therefore, compared with the multiplicative approach, the additive approach is more suitable for hybrid filler systems. The aforementioned model prediction is based on the following assumptions: the polymer is unaffected by the presence of the filler (e.g., no change in polymer crystallinity and no change in filler orientation), the matrix and the filler are isotropic and strongly bonded, and no filler–filler interactions or agglomerations occur [31]. The morphology of the composite films in this work is, nevertheless, far more complex than morphologies based on these assumptions. Moreover, the distribution of fillers in the surface of casting films usually differs from the distribution at the center. However, the simplified model presented here enables the behavioral prediction of an ideal structure and filler distribution and elucidates the basic role of the fillers in thermally conductive polymer composite films.

## 4. Conclusions

PI-based thermally conductive composite materials were fabricated using anisotropic BN, spherical AlN, and Al_2_O_3_ particles. Among them, the PI/BN composite had outstanding thermal conductivity in the through- and in-plane directions. In this composite, some amount of Al_2_O_3_ and AlN were added to the binary filler. In the case of the through-plane direction, the hybrid filler system shows lower thermal conductivity than the PI/BN composite, whereas in-plane thermal conductivity was enhanced because the BN particles were horizontally aligned via the binary filler. The maximum thermal conductivity value along the in-plane direction was 4.091 W/m∙K for the film containing 30 wt % of PI/BN + AlN filler, whereas the value of the PI/ BN composite was 3.371 W/m∙K. These experimental results were compared with the Lewis–Nielsen and modified Lewis–Nielsen theoretical prediction models. The PI/BN composite corresponded better with the theoretical model than with the Al_2_O_3_ and AlN composites. However, these theoretical predictions are only applicable to the single-filler system. Therefore, the additive and multiplicative approaches were applied to PI/BN + Al_2_O_3_ and PI/BN + AlN composites. This has not been reported previously for the prediction of a binary filler system. As a result, the additive approach shows a better fit with experimental results, whereas the multiplicative approach overestimates, especially for a high filler concentration.
